# Evaluating cross-cutting opportunities for dog-mediated rabies control: a scoping review

**DOI:** 10.3389/fmicb.2025.1473929

**Published:** 2025-06-18

**Authors:** R. Tidman, A. Larkins, A. Auplish, C. Benfield, S. Cleaveland, Anna S. Fahrion, V. Leopardi, R. Ghimire, K. Morucci, S. Sila, R. Castillo-Neyra, A. Okello

**Affiliations:** ^1^Department of Science, World Organisation for Animal Health, Paris, France; ^2^School of Medical, Molecular and Forensic Sciences, Murdoch University, Perth, WA, Australia; ^3^Centre for Biosecurity and One Health, Harry Butler Institute, Murdoch University, Perth, WA, Australia; ^4^Food and Agriculture Organization of the United Nations, Rome, Italy; ^5^School of Biodiversity, One Health and Veterinary Medicine, College of Medical, Veterinary and Life Sciences, University of Glasgow, Glasgow, United Kingdom; ^6^Friedrich-Loeffler-Institut, Greifswald, Germany; ^7^Department of Biostatistics, Epidemiology and Informatics, University of Pennsylvania, Philadelphia, PA, United States; ^8^Vetsuisse Faculty, University of Zürich, Zürich, Switzerland; ^9^Australian Centre for International Agricultural Research, Canberra, ACT, Australia; ^10^College of Medicine and Veterinary Medicine, University of Edinburgh, Edinburgh, United Kingdom

**Keywords:** dog-mediated rabies, rabies elimination, zero by 30, One Health, United Against Rabies, neglected tropical diseases, cross-cutting, integrated approaches

## Abstract

**Background:**

Despite rabies being preventable, the disease continues to be under-prioritised and under-resourced, competing with other human and animal health diseases and socio-political agendas. The control of dog-mediated human rabies is a model of One Health operationalisation, and the One Health approach is core to the “Zero by 30” goal. There have been several opportunities proposed and/or piloted for the integration of rabies with other disease control efforts and interventions, in line with this One Health approach.

**Methods:**

A scoping review was conducted, following PRISMA guidelines, to summarise the nature and outcomes of cross-cutting approaches that have been applied to understand the opportunities available and evaluate the contexts in which such approaches can add value. Studies were included which demonstrated evidence describing an approach focused on dog-mediated rabies control and another health or development intervention affecting humans, animals, or the environment. In addition to the literature review, expert consultations were conducted to inform the development of recommended criteria or questions to consider when exploring cross-cutting or integrated approaches.

**Results:**

Records were mapped against the WHO NTD roadmap cross-cutting approach categories to help classify the evidence. Thirteen records in total were included in the review, with two of these records including aspects of multiple categories. Two records included evidence of planning and programme management; eleven records included evidence of activities or approaches associated with implementation and three records included evidence related to monitoring and evaluation, specifically surveillance. Insights from expert consultations complemented the available literature and led to the development of key criteria to consider when exploring cross-cutting approaches for rabies control.

**Conclusion:**

Integrated or cross-cutting approaches can offer the opportunity to enhance and build upon common and existing delivery platforms for health services and maximise the impact of limited resources. However, integrated approaches could have detrimental effects and their implementation requires careful consideration. Further evidence is needed to understand where cross-cutting approaches can be effective, sustainable, and scalable to support the agenda to end human deaths from dog-mediated rabies by 2030.

## Introduction

Rabies continues to kill an estimated 59,000 people each year, 40% of whom are children living in Asia and Africa (Hampson et al., 2015). Approximately 99% of human rabies cases are caused by bites from domestic dogs, and the disease imposes a heavy economic burden of US$8.6 billion per year (Hampson et al., 2015; World Health Organization et al., 2018). Despite rabies being preventable through increased awareness, improved access to post-exposure prophylaxis and mass dog vaccination, and an established Global Strategic Plan to end human deaths from dog-mediated rabies by 2030, the disease continues to be under-prioritised and under-resourced, outcompeted by other human and animal health diseases and socio-political agendas (World Health Organization et al., 2018).

The elimination of dog-mediated human rabies is a model of One Health operationalisation—requiring collaboration between human and animal health sectors to effectively implement rabies control and elimination programmes (Tidman et al., 2022). The One Health approach is core to “Zero by 30: the Global Strategic Plan to end human deaths from dog-mediated rabies by 2030” (Zero by 30) (World Health Organization et al., 2018). Building on this strategy, the United Against Rabies (UAR) Forum was established by Food and Agriculture Organization of the United Nations (FAO), World Health Organization (WHO), and World Organisation for Animal Health (WOAH, founded as OIE) in 2020 to implement its objectives and bring together a diverse range of stakeholders to work together towards this global goal (Tidman et al., 2022). One of the activities within the UAR Forum focused on exploring and critically evaluating cross-cutting opportunities for rabies control wherein rabies control is integrated with other interventions or policy frameworks. Guidelines already exist to help countries implement One Health plans, including the WHO road map for neglected tropical diseases (NTDs), and the Quadripartite One Health Joint Plan of Action (Tidman et al., 2023; World Health Organization, 2020; Food and Agriculture Organization of the United Nations et al., 2022), and the aim of this activity was to provide further support to countries to integrate rabies control within the wider One Health agenda.

There have been several opportunities suggested for the integration of rabies with other disease control efforts and interventions, including for snake-bite envenomation (Scott et al., 2021), parasitic diseases (such as soil-transmitted helminths and echinococcosis) in humans and animals (Lankester et al., 2019; El Berbri et al., 2020), and combined vaccination campaigns of dogs and livestock (such as foot and mouth disease and peste des petits ruminants) (Food and Agriculture Organization of the United Nations, 2024). Integrated or cross-cutting approaches can potentially offer the opportunity to enhance and build upon common and existing delivery platforms for health services, improve the cost-effectiveness and coverage of health interventions, and strengthen cross-sectoral coordination mechanisms. Capturing these opportunities may have wide reaching co-benefits including improving pandemic preparedness and response, and achieving universal health coverage (World Health Organization, 2020). However, there is a possibility that such integrated approaches may have detrimental effects, including additional costs and delivery challenges, negative perceptions, and may risk spreading already scarce resources too thinly. Thus, the appropriateness and implementation of integrated approaches will vary according to sociocultural, economical, epidemiological and geographical context (World Health Organization, 2020). There is a need to summarise the nature and outcomes of the approaches that have been applied in the past to understand the possible opportunities available and evaluate the contexts in which these approaches can add value.

This review was undertaken by a UAR Forum workstream entitled “Cross-cutting opportunities for rabies control.” The objective of this workstream was to provide an evidence base to inform the development of any future guidance and recommendations on cross-cutting approaches for dog-mediated rabies control. UAR Forum members conducted a scoping review of available evidence of integrated or cross-cutting approaches, aiming to identify strengths, weaknesses, opportunities, and threats of such approaches. This publication presents the results of this scoping review, with plans for the UAR Forum to work with stakeholders to further develop guidance and recommendations to support countries with the planning, implementation and monitoring and evaluation of cross-cutting approaches in future.

## Method

### Search strategy and selection criteria of published scientific literature

A scoping review was performed following the Preferred Reporting Items for Systematic Reviews and Meta-Analyses Extension for Scoping Reviews (PRISMA-ScR) guidelines (Tricco et al., 2018).

UAR Forum workstream participants searched electronic databases, including PubMed, Scopus, and Web of Science for published reports of cross-cutting and integrated approaches for rabies control. Search terms included “rabies,” “combined,” “integrated” and “cross-cutting.” The terms were searched in titles and abstracts and Boolean logic operators “AND” and “OR” and wildcards (e.g., “integrat*”) were used. Where possible, MeSH terms were applied. A full list of queries can be found in the Supplementary material. The search was carried out between April and May 2022. No publication date restriction was applied to the search, and no language restrictions were set, although the initial search terms were in English, with search terms then translated to Spanish and the search strategy repeated. Spanish was selected due to the success of rabies control in the Americas, and the well-established Rabies in the Americas network that has facilitated the sharing of scientific knowledge on rabies since 1990. Further translation of search terms into additional languages was considered by the workstream but was decided against due to the limited publications identified using English and Spanish search terms.

Identified records were initially imported into Zotero (Corporation for Digital Scholarship, 2022) for de-duplication, followed by title and abstract screening using Rayyan software (Mourad et al., 2016). Screening of title and abstracts were based on two screening questions: does the evidence consider dog-mediated rabies; and does the evidence describe an approach including dog-mediated rabies control and another health or development intervention affecting humans, animals, or the environment? A minimum of two reviewers screened each title/abstract for inclusion, with any conflicts or uncertainty resolved by an additional reviewer who had not already screened the study in question. Records that met the eligibility criteria during title and abstract screening were exported into Excel for full-text screening, using the same two screening questions. Full-text records were excluded if they did not meet the inclusion criteria, were unavailable, were review articles, or lacked a description of an integrated or cross-cutting approach involving dog-mediated rabies and another health or development issue (e.g., the record instead was simply a call to action for integrated approaches, or the record identified integrated approaches across sectors but only for dog-mediated rabies with no other health or development issue). Records were not assessed for quality. A minimum of two reviewers screened each full text for inclusion, with conflicts or uncertainty resolved in discussion with additional reviewers.

### Additional information sources

The search strategy was complemented by requests to members of the UAR Forum in 2022 to provide any literature or experience that they may have of integrated or cross-cutting approaches. An additional call for input was circulated in the UAR Forum May 2023 newsletter, which was circulated to 1712 individuals (with potential to be forwarded to additional individuals) and promoted on UAR Forum social media networks.

Due to the success of rabies control programmes in the Americas region, abstracts from the Rabies in the Americas conferences dating from 1991 to 2022 were also screened, and UAR Forum members based in this region reached out informally to their networks to request any further grey literature or anecdotal evidence.

### Expert consultation

This topic was explored further in the “Integrative Interventions Across Neglected Tropical Diseases to Support Sustainable Public Health Policy” workshop, held at the University of Surrey, United Kingdom in May 2024. To complement the findings from the literature review, several UAR Forum members participated in discussions with wider NTD experts to inform the development of recommended criteria or questions to consider when exploring cross-cutting or integrated approaches. The findings from these expert consultations are presented alongside the literature review findings in the Results section.

## Results

### Literature review

**Figure 1:**
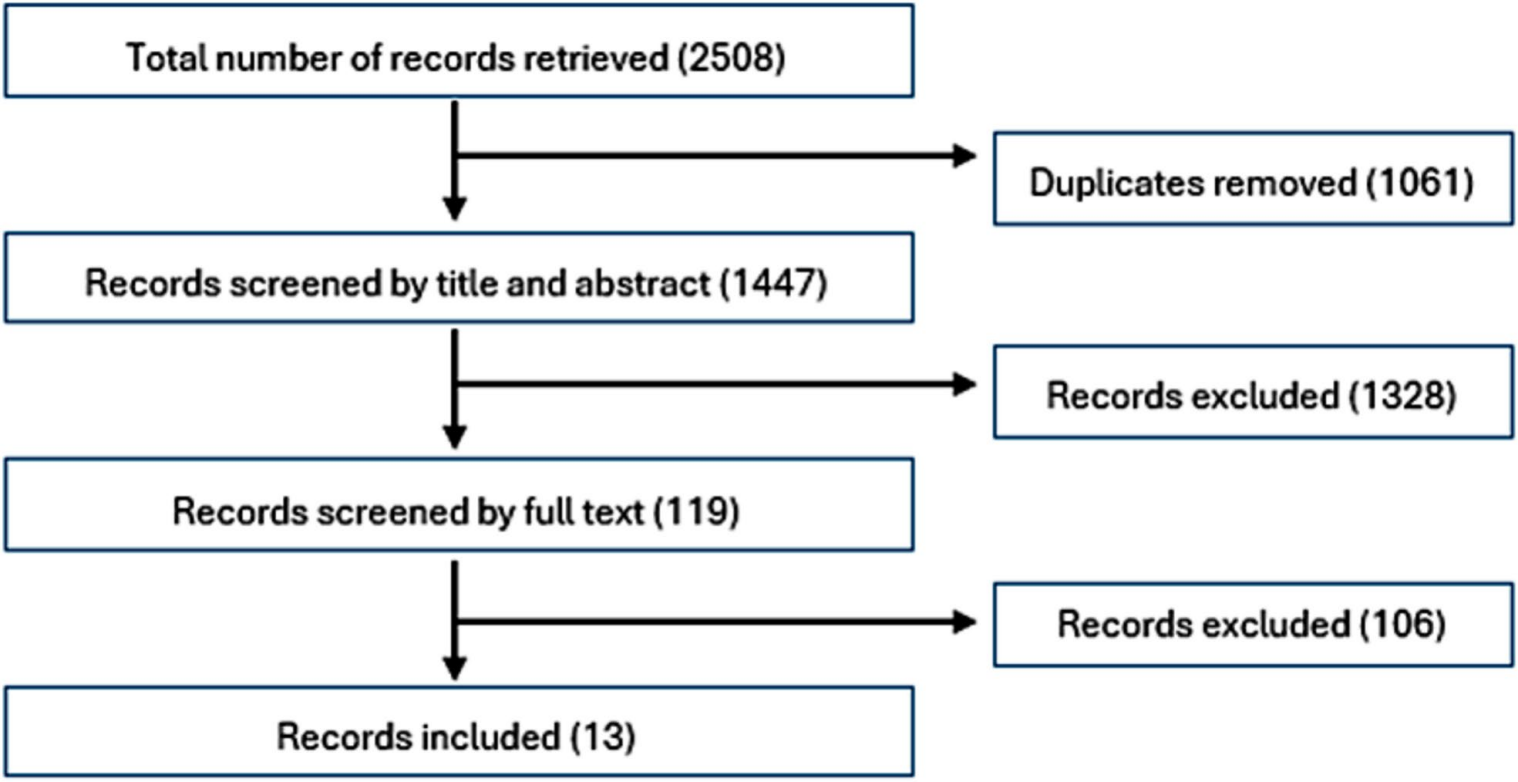
PRISMA flowchart for record selection.

The search strategy yielded a total of 2,508 records. After screening of titles and abstracts, 119 records were kept for full-text screening. No additional published or grey literature was identified by the informal networks that had not already been identified by the search strategy, or by knowledge from the authors. A final set of 13 records met the inclusion criteria and are part of this review (Figure 1). The reduction from 2508 to 1447 records was due to the removal of duplicates, with further reduction from 1447 to 119 records due to the removal of non-relevant studies based on title and abstract screening. The further reduction to 13 records resulted from the application of the strict inclusion criteria listed above.

The UAR Forum workstream initially aimed to extract data relating to the study period and location, the study scale (pilot or proof-of-concept, local/sub-national, national, regional), the target human and dog population of the study, what sort of approach was used, the diseases being targeted, the actors involved, who was responsible for funding the project/programme, the benefits and challenges of the approach, whether the approach had been effective and sustainable, and whether the approach was shown to be economical or cost-effective. However, few records included information on these categories, and many simply reported the existence or trial of a cross-cutting approach, without providing more detailed information. Data extraction therefore focused instead on strengths/benefits and weaknesses/challenges of the cross-cutting approach reported by the primary authors of each record. Extracted data has been summarised and grouped according to categories defined in the WHO NTD roadmap (World Health Organization, 2020) (planning and programme management; implementation; monitoring and evaluation) (Table 1).

**Table 1:** Summarised data extracted from records giving examples of cross-cutting approaches for rabies control and their scale of approach, strengths/benefits, and weaknesses/challenges.

WHO NTD roadmap category of cross-cutting approach (World Health Organization, 2020)	Number of records	Description of approach	Scale of approach	Strengths or benefits	Weaknesses or challenges	References
Planning and programme management	1	Mapping of multiple NTDs (lymphatic filariasis, onchocerciasis, human rabies, schistosomiasis, trachoma)	Regional (Latin America and Caribbean)	Allowed identification and geographical visualisation of where diseases are present and overlapping, informing the possibilities for interprogrammatic and intersectoral approaches, and potential for cross-border activities	Not described	Schneider et al. (2011)
	1^a^	Data management and mapping of zoonotic diseases, venomous animal injuries and vector-borne disease in Brazil	Local level/sub-national (Brazil)	Increased number of investigations, disease notifications and gathering of more robust data for each of the included diseases Integrated digital surveillance allowed efficiently updated information to inform decision makers and improve response time to disease notifications	Not described	de Souza Leandro et al. (2021)
Implementation	3	Integrated prevention/treatment delivery: zoonotic diseases [soil-transmitted helminthiases (STH) and dog-mediated rabies in Tanzania (Lankester et al., 2019; Davis et al., 2022); rabies, leishmaniasis and cystic echinococcosis in Morocco (El Berbri et al., 2020)]	Local level proof-of-concept or pilot studies (Tanzania, Morocco)	Positive community perception in terms of receiving “two for one” health intervention, saving time and effort, and reducing participant costs with integrated STH and dog-mediated rabies interventions in Tanzania (Lankester et al., 2019; Davis et al., 2022) Cost saving with cost per dose was lower for integrated delivery in Tanzania, with 33% reduction in cost per dose reported for STH, and 16% lower cost per rabies vaccination (Lankester et al., 2019). Integrated costs estimated to be 30% lower for combined costs for single disease interventions in Morocco (El Berbri et al., 2020) Time-saving with 33% less time for a single person and a dog to attend a combined event (for deworming and dog vaccination) in Tanzania than to attend two separate events (Lankester et al., 2019) Building One Health capacity—integrated interventions catalysed effective collaboration between human and animal health workers (Lankester et al., 2019)	Negative community perception in terms of integrated human and animal health interventions being “unhygienic” or “difficult” (Lankester et al., 2019; Davis et al., 2022) Potential for one intervention to compromise outcome for the other, i.e., 60% of respondents in Tanzania would prioritise having children dewormed over vaccinating dogs (Lankester et al., 2019) Potential for intensity of multiple treatments may dissuade owners from seeking continued intervention (e.g., repeated annual rabies vaccinations for dogs) (El Berbri et al., 2020)	Lankester et al. (2019), El Berbri et al. (2020), and Davis et al. (2022)
	3	Integrated prevention/treatment: animal diseases [rabies, cattle foot and mouth disease and contagious bovine pleuropneumonia in Namibia and Angola (Athingo et al., 2020); rabies and contagious bovine pleuropneumonia in Kenya (Coleman, 1999); rabies and peste des petits ruminants (PPR) in Guinea, Liberia and Sierra Leone] (Alpha, 2022)	Local level proof-of-concept or pilot studies (Namibia and Angola, Kenya, Sierra Leone)	Cost-savings associated with sharing of resources in rabies and PPR integrated interventions in Guinea, Liberia and Sierra Leone (e.g., vaccine storage facilities, vaccination teams, vehicles) (Alpha, 2022) Building One Health capacity: integrated approaches reported to improve collaboration between sectors (Food and Agriculture Organization of the United Nations, 2024)	Lower dog vaccination coverage when delivered with cattle CBPP vaccine than when delivered through house-to-house visits (Coleman, 1999)	Athingo et al. (2020), Coleman (1999), and Alpha (2022)
	3	Joint awareness-building and community education [rabies, leishmaniasis, cystic echinococcosis, brucellosis and bovine tuberculosis in Morocco (Ducrotoy et al., 2015); rabies and leprosy in Philippines (Macabihag, 2018); rabies and PPR in Guinea, Liberia and Sierra Leone (Alpha, 2022)]	Local level proof-of-concept or pilot studies (Morocco, Philippines, Guinea, Liberia and Sierra Leone)	Improved community participation, awareness and community engagement reported from joint rabies and PPR awareness campaigns (Food and Agriculture Organization of the United Nations, 2024) Greater community interest generated for under-recognised diseases when messaging for these were associated with higher-priority diseases, than if diseases were addressed separately (Ducrotoy et al., 2015)	Integrated messaging for multiple zoonoses resulted in information overload for communities when transmission pathways did not overload (Ducrotoy et al., 2015) Potential for health messaging of multiple diseases (e.g., rabies, leishmaniasis, cystic echinococcosis) all in one reservoir host (dogs) to exacerbate negative perceptions of dogs (Ducrotoy et al., 2015)	Ducrotoy et al. (2015), Macabihag (2018), and Alpha (2022)
	1^a^	One Health integrated approach for wildlife conservation of Ethiopian wolves in Ethiopia	Local/sub-national (Ethiopia)	Improved strategies for outbreak response, disease control, surveillance and monitoring, and conservation in Ethiopian wolves	Not described	Randall et al. (2006)
	1^a^	Health care worker training/rapid response systems (zoonotic diseases, venomous animal injuries, vector-borne disease in Brazil)	Local level/sub-national (Brazil)	Increased number of investigations, disease notifications and gathering of more robust data for each of the included diseases Single health agent was able to investigate multiple diseases, rather than multiple visits from multiple health agents, resulting in health agents being better received by communities Trained, flexible One Health agents that could efficiently assist in surveillance and response for other diseases when required (e.g., SARS-Cov-2)	Not described	de Souza Leandro et al. (2021)
	1	Laboratory diagnosis (laboratory network for NTDs which is intended to support surveillance systems, provide laboratory confirmatory services, external quality assessment, and training in Philippines)	National (Philippines)	Not described	Not described	Leonardo et al. (2020)
Monitoring and evaluation	3^a^	Surveillance [adoption of digital surveillance approaches and active surveillance in Brazil (de Souza Leandro et al., 2021); epidemiological surveillance system for leptospirosis, brucellosis, rabies, tuberculosis, plague in Ecuador (Torres et al., 2021); monitoring of population dynamics and rabies in Ethiopian wolves in Ethiopia (Randall et al., 2006)]	Local/sub-national (Brazil and Ethiopia); national (Ecuador)	Increased number of investigations, disease notifications and gathering of more robust data for each of the included diseases (de Souza Leandro et al., 2021) Integrated digital surveillance allowed efficiently updated information to inform decision makers and improve response time to disease notifications (de Souza Leandro et al., 2021) Comprehensive visits from One Health agents resulted in improvement of active surveillance, and a resultant reduction in time to respond to reported notifications (de Souza Leandro et al., 2021)	Not described	de Souza Leandro et al. (2021), Randall et al. (2006), and Torres et al. (2021)

a Records that reported aspects of more than one WHO NTD roadmap category of cross-cutting approach. One record included aspects of “Planning and programme management,” “Implementation,” and “Monitoring and evaluation” (de Souza Leandro et al., 2021). One record included aspects of “Implementation” and “Monitoring and evaluation” (Randall et al., 2006).

### Planning and programme management

Two records (Schneider et al., 2011; de Souza Leandro et al., 2021) highlighted integrated or cross-cutting approaches associated with planning and programme management which included rabies, both of which focused on the Americas. Mapping of selected neglected tropical diseases in the Americas helped to identify areas where multiple diseases overlapped, and where integrated and intersectoral approaches could be impactful, but noted that mapping needed to be refined to community level for proper planning and decision-making (Schneider et al., 2011). Integrated health care worker training, rapid response systems and digital integration of disease mapping in Brazil were shown to result in more effective and efficient decision-making, and the provision of guidelines for specific, effective and timely response to cases/disease outbreaks (de Souza Leandro et al., 2021), although there was no explicit evidence stated as to whether this improved for alerting communities.

### Implementation

Eleven records (Lankester et al., 2019; El Berbri et al., 2020; de Souza Leandro et al., 2021; Davis et al., 2022; Athingo et al., 2020; Coleman, 1999; Ducrotoy et al., 2015; Macabihag, 2018; Alpha, 2022; Randall et al., 2006; Leonardo et al., 2020) included in this review described activities or approaches associated with implementation, and these included sub-categories (as defined by the WHO NTD roadmap) of integrated prevention/treatment delivery, joint awareness-building and community education, One Health integrated approaches for wildlife conservation, healthcare worker training and rapid response systems, and laboratory diagnosis. With the exception of one record describing One Health training and disease investigation in Brazil (de Souza Leandro et al., 2021), and one record discussing integrated NTD laboratory diagnosis in the Philippines (Leonardo et al., 2020), all other records exploring cross-cutting approaches for implementation were proof-of-concept projects, or projects conducted at a local or sub-national level.

The key strengths and benefits described for cross-cutting approaches for implementation activities included: improved community perception, awareness and engagement (Lankester et al., 2019; de Souza Leandro et al., 2021; Davis et al., 2022; Ducrotoy et al., 2015; Alpha, 2022); cost and time-saving for integrated interventions when compared to separate interventions for single diseases (Lankester et al., 2019; El Berbri et al., 2020; Alpha, 2022); improved disease notification and response time (de Souza Leandro et al., 2021); improved strategies for wildlife conservation (Randall et al., 2006); and improved intersectoral collaboration between human and animal health sectors and the building of One Health capacity that could then be flexibly used to address other disease threats (Lankester et al., 2019; de Souza Leandro et al., 2021; Alpha, 2022).

Weaknesses and challenges were seldomly reported, but included negative community perception about joint human and animal health interventions (with some community members reporting concerns about the intervention being “difficult” or “unhygienic”) (Lankester et al., 2019; Davis et al., 2022), logistical challenges (e.g., participants unable to transport both dogs and children to central locations for treatment) (Davis et al., 2022). There was the possibility that interventions for multiple diseases could be detrimental to control efforts for one disease—lower dog vaccination coverage for rabies was achieved when delivered with cattle CBPP vaccine than when delivered through house-to-house visits in Kenya (Coleman, 1999), and when faced with logistical challenges participants noted that they would be more likely to choose de-worming treatment for their children instead of rabies vaccination for dogs, despite rabies being the more severe health threat (Lankester et al., 2019). One record noted that joint health education messaging also resulted in information overload for communities when transmission pathways did not overlap, and when it did overlap (as in the case for diseases which are all dog-mediated such as rabies, leishmaniasis and cystic echinococcosis) there was the potential for combined health messaging to exacerbate negative perceptions of dogs (Ducrotoy et al., 2015). Another record noted that there was a possibility that the intensity of providing multiple treatments for dogs dissuaded owners from seeking more regular interventions such as repeated annual rabies vaccination for dogs (El Berbri et al., 2020).

Few records reported the long-term effectiveness or sustainability of these approaches. Two records included an economic analysis, highlighting favourable results for cross-cutting approaches with reduced costs due to shared transport, combined community announcements and staff salaries. There was a 33% lower cost reported per deworming dose for children, and a 16% lower cost per rabies vaccination for dogs in Tanzania (Lankester et al., 2019), and a 30% lower cost for combined interventions (rabies, leishmaniasis and cystic echinococcosis) in Morocco (El Berbri et al., 2020) than if these interventions had been implemented separately.

### Monitoring and evaluation

Three records (de Souza Leandro et al., 2021; Randall et al., 2006; Torres et al., 2021) included descriptions of surveillance, but only one of these described strengths and benefits, which included improved active surveillance with increased number of disease notifications and gathering of more robust data, and efficient updating of information for multiple diseases which informed decision making and improved response time (de Souza Leandro et al., 2021).

### Expert consultation

The findings from expert consultations with wider NTD experts informed the development of key questions that stakeholders are recommended to consider when exploring cross-cutting or integrated approaches for rabies control (Box 1).

BOX 1 **Questions to consider when exploring cross-cutting or integrated approach for rabies**

- Are both diseases priority pathogens?
- Is there a common or shared host, vector, or transmission pathway?
- Are there similar programmatic goals (e.g., control versus elimination)?
- Are the diseases of similar severity?
- Is there a similar geographical spread/overlap of the diseases?
- Are similar interventions required for disease control?
- Are similar diagnostic methods used?
- Are there similar delivery requirements for interventions for both (e.g., cold chain for vaccines)?
- Does an integrated or cross-cutting approach improve efficiency in one or both diseases?
- What type/level of community engagement is needed for both?

Further work is anticipated by the UAR Forum and wider NTD networks to refine and test these recommendations (through identification of case studies, interviews with stakeholders directly involved in these initiatives) as to whether a cross-cutting approach should be explored further.

The UAR Forum includes One Health and rabies experts who are available to support stakeholders with planning, implementing, and monitoring and evaluating cross-cutting and integrated approaches for rabies control and recommends that any such operational research focused on this area include transparent reporting of the benefits, challenges, economics, and long-term sustainability of such approaches. Several international guidance documents exist to help countries identify opportunities or implement One Health approaches, including “A tripartite guide to addressing zoonotic diseases in countries” (The Food and Agriculture Organization of the United Nations et al., 2019), the WHO NTD roadmap (World Health Organization, 2020) and “A guide to implementing the One Health Joint Plan of Action at national level” (World Health Organization et al., 2023).

The UAR Forum encourages stakeholders to share or publish any evidence on cross-cutting approaches, to add to this evidence base, so that this knowledge and experience can be used to inform and adapt integrated or cross-cutting approaches for dog-mediated rabies elsewhere, and Box 2 includes specific recommendations to strengthen the evidence base. Stakeholders interested in engaging with the UAR Forum further on this topic are encouraged to visit www.unitedagainstrabies.org, or email globalrabiescoordinator@woah.org.

BOX 2 **Filling gaps to advance integrated approaches to eliminate dog-mediated human rabies**

As this paper highlights, there are gaps in the current evidence base to provide robust guidance. The following recommendations are aimed at strengthening the evidence base:

- Expand and diversify publications to include non-research integrative programmes.
- Increase support and funding for research in this area.
- Promote the publication of both positive and negative outcomes related to this subject to prevent publication bias.
- In reports and publications, provide scalability assessments, economic evaluations, strengths, benefits, weaknesses, and challenges.

## Discussion

Cross-cutting approaches present an opportunity for enhancing and building upon common and existing delivery platforms for health services to provide potentially cost-effective and innovative ways of disease prevention and control, in line with the One Health approach. Despite the globally stated prioritisation of One Health, integrated and systems-based approaches, and calls for cross-cutting approaches for NTDs such as rabies, there is limited published evidence either in peer-reviewed journals or the grey literature for cross-cutting or integrated approaches for rabies control apart from examples such as integrated bite case management (IBCM) and rabies education programmes. While these topics certainly dealt with One Health and cross sectoral collaboration, they focused solely on dog-mediated rabies and were excluded for the purpose of this review.

Overall, the benefits reported in this review align with those highlighted in key global policy documents such as the WHO NTD roadmap and One Health Joint Plan of Action (World Health Organization, 2020; Food and Agriculture Organization of the United Nations et al., 2022). However, few of the records reporting these benefits outline whether these approaches had potential to scale up to broader sub-national, national, or regional programmes, and still demonstrate these benefits. What may be effective in a proof-of-concept study may be logistically and financially challenging to integrate and implement at a larger scale (Tsing, 2012). Alternatively, it may be that cross-cutting approaches particularly for the planning and programme management and monitoring and evaluation categories, are a well-established as part of regular regional, national, or sub-national disease control programmes, but are not necessarily reported in the literature. The lack of published examples could reflect challenges or lack of incentives in documenting and disseminating experiences rather than an absence of such initiatives. Strengthening mechanisms for reporting and knowledge sharing could help address this gap.

While some of the records presented promising results in terms of cost-effectiveness (Lankester et al., 2019; El Berbri et al., 2020), very few included a robust cost-benefit analysis of the reported cross-cutting approaches, or any evaluation of long-term sustainability. Economic evaluations producing quantitative outcomes for the costs and benefits across involved sectors can be used to determine whether there is a positive return on the investment and strengthen the case for investing in an integrated or cross-cutting approach. However, it must be remembered and highlighted to decision-makers that cost–benefit analyses only consider monetary values. They do not capture other important measures such as improved animal welfare or human health. Economic evidence is a key factor for demonstrating the added value of cross-cutting approaches and can be used to support decisions for resource allocation. Strengthening the evidence-base will be critical to advocate for policy makers to adopt, implement and endorse such approaches.

Further important potential benefits of integrated strategies include improvements in relationships, communication and trust between sectors, but these were mentioned by only three records. While assessment of these factors is often more challenging than evaluation of quantitative metrics, future studies should attempt to capture these benefits. Trust has been identified as a critical factor in determining the outcome of human and animal health interventions (Vinck et al., 2019; Devine et al., 2021; Auty et al., 2021) and lack of familiarity with methods of assessment should not be a reason for precluding their inclusion in the design of future studies.

There were fewer weaknesses, threats and challenges reported for cross-cutting approaches compared to single disease programmes. It is difficult to know if this is a true reflection of stakeholders’ experience of planning, implementing, or evaluating these approaches, or whether instead there may be a bias towards reporting more positive outcomes (general publication bias). Similarly, it may be that integrated approaches that are not perceived as beneficial or successful are not promoted or published. However, it is critical that barriers in these approaches are transparently shared and reported, for other stakeholders to draw lessons from the experiences of others and identify ways in which approaches can be adapted to either avoid or overcome these challenges.

There were several limitations with this review. Firstly, while efforts were made to include languages other than English in our search of both published and grey literature, there may be evidence in additional languages that has not been captured resulting in possible language bias. Secondly, the search terms and screening strategy used may also have resulted in key literature being missed, particularly in the categories of “planning and programme management”, and “monitoring and evaluation,” as our search strategy was more biased towards approaches involving “implementation.” Thirdly, as much of the data was qualitative and subjective, there was a degree of inference made by the reviewers in terms of defining which category aligned most closely with each cross-cutting approach, and in extracting data that described strengths and weaknesses of each approach. Fourthly, by screening articles based on whether they described a real-world or a modelled case study that had been carried out, we also screened out any literature that may highlight where potential opportunities exist for integrated control for rabies, but had not been studied, and these data could be valuable for informing future research. Finally, the workstream elected to exclude records that were clearly outlining a cross-cutting or integrated approach but still focused only on dog-mediated rabies (e.g., records that included integrated bite case management, rabies education programmes, or rabies and dog population management). These are all well-documented aspects of effective dog-mediated rabies control programmes, and it was the aim of the workstream to explore broader aspects of cross-cutting approaches. However, these records may well have included valuable information that could be applied to broader cross-cutting approaches for dog-mediated rabies.

While integrated and cost-cutting approaches may offer unexplored opportunities that could accelerate progress towards the “Zero by 30” goal, it is crucial that they do not dilute the focus on rabies control. In high-burden settings, maintaining targeted, well-resourced rabies elimination programmes must remain a priority, with cross-cutting strategies serving as a complementary tool rather than a replacement for direct intervention efforts. This is especially true in regions such as Africa and Asia, where the burden of dog-mediated rabies remains high and targeted mass dog vaccination and community engagement are likely to offer the greatest impact. The application of integrated and cross-cutting approaches should be carefully tailored to the needs and priorities of individual settings. A one-size-fits-all strategy is unlikely to be effective; in regions where rabies remains a critical public health threat, dedicated, well-funded rabies control programmes must be the cornerstone of elimination efforts, with integrated strategies considered only when they demonstrably enhance programme efficiency, reach, or sustainability without compromising core programmatic goals.

## Conclusion

The control of dog-mediated rabies remains one of the best available models for the implementation of the One Health approach on the ground. Despite the growing global momentum for systems-based and cross-cutting (One Health) approaches for prevention and control, and the number of opportunities for the integration of rabies with other disease control interventions, evidence of its sustainable success is scarce. Integrated and cross-cutting approaches have the potential to improve community engagement, maximise the use of limited resources, and to build flexible One Health capacity. Yet, there is also a possibility that integrated approaches may be detrimental to disease control efforts or be unsustainable or unscalable manner, therefore using such an approach requires careful consideration, which is an important conclusion of this review.

While the current evidence base is too limited to develop robust guidance or frameworks for specific integrated or cross-cutting approaches for rabies control and other health or development issues in humans, animals, or the environment, this review does highlight gaps where further evidence is needed, as well as positive examples which could be explored by other countries. Addressing gaps in published literature through improved documentation and knowledge-sharing will be critical for informing future policy and programme design.

While this review begins to contribute to the growing evidence to support researchers and decision makers, further evidence is required to fill the gaps to advance integrated and cross-cutting approaches that are most appropriate and impactful for the control of dog-mediated rabies. Exploring untapped opportunities could accelerate progress towards the “Zero by 30” goal, emphasizing evidence-based cross-cutting interventions for effective rabies control and elimination.

## Data Availability

The original contributions presented in the study are included in the article/Supplementary material, further inquiries can be directed to the corresponding author/s.
