# Supplementary material for: Evaluating cross-cutting opportunities for dog-mediated rabies control: a scoping review
Source: Front Microbiol. 2025 Jun 18;16:1473929. doi: 10.3389/fmicb.2025.1473929 (PMC12223422; doi:10.3389/fmicb.2025.1473929)
Supplement: Supplementary file 1 [file Table_1.docx]

# Supplementary data

The initial English search was completed by UAR Forum workstream members by applying the following queries:

- PubMed: *rabies[MeSH Terms] AND (integrat*[Title/Abstract] OR join*[Title/Abstract] OR combin*[Title/Abstract] OR cross-cutting[Title/Abstract] OR crosscutting[Title/Abstract] OR (cross AND cutting) [Title/Abstract])*
- Web of Science: *TS=("rabies" AND (integrat* OR join* OR combin* OR cross-cutting OR crosscutting OR (cross AND cutting)))]*
- Scopus: *TITLE-ABS-KEY (rabies AND (integrat* OR join* OR  combin*  OR  cross-cutting OR crosscutting OR  (cross AND  cutting)))*
